# Neutrophils as Regulators and Biomarkers of Cardiovascular Inflammation in the Context of Abdominal Aortic Aneurysms

**DOI:** 10.3390/biomedicines9091236

**Published:** 2021-09-16

**Authors:** Johannes Klopf, Christine Brostjan, Christoph Neumayer, Wolf Eilenberg

**Affiliations:** Department of General Surgery, Division of Vascular Surgery, Medical University of Vienna, University Hospital Vienna, 1090 Vienna, Austria; johannes.klopf@meduniwien.ac.at (J.K.); christine.brostjan@meduniwien.ac.at (C.B.); christoph.neumayer@meduniwien.ac.at (C.N.)

**Keywords:** neutrophils, cardiovascular diseases, biomarkers, abdominal aortic aneurysm, neutrophil granules, myeloperoxidase, neutrophil elastase, neutrophil extracellular traps (NETs), matrix metalloproteinase (MMP), inflammation

## Abstract

Neutrophils represent up to 70% of circulating leukocytes in healthy humans and combat infection mostly by phagocytosis, degranulation and NETosis. It has been reported that neutrophils are centrally involved in abdominal aortic aneurysm (AAA) pathogenesis. The natural course of AAA is growth and rupture, if left undiagnosed or untreated. The rupture of AAA has a very high mortality and is currently among the leading causes of death worldwide. The use of noninvasive cardiovascular imaging techniques for patient screening, surveillance and postoperative follow-up is well established and recommended by the current guidelines. Neutrophil-derived biomarkers may offer clinical value to the monitoring and prognosis of AAA patients, allowing for potential early therapeutic intervention. Numerous promising biomarkers have been studied. In this review, we discuss neutrophils and neutrophil-derived molecules as regulators and biomarkers of AAA, and our aim was to specifically highlight diagnostic and prognostic markers. Neutrophil-derived biomarkers may potentially, in the future, assist in determining AAA presence, predict size, expansion rate, rupture risk, and postoperative outcome once validated in highly warranted future prospective clinical studies.

## 1. Introduction

Neutrophils are the most abundant type of circulating leukocytes in healthy adults and play a central role during the innate immune response [1]. These diverse cells are not limited to the elimination of pathogens, but further act as mediators with specialized functions in health and inflammation [2]. Beside the manifold strategies to execute the tasks of the non-specific, innate immune response including phagocytosis, cytokine secretion, and degranulation, neutrophils also contribute to chronic inflammatory conditions by the release of reactive oxygen species (ROS) and neutrophil-derived microvesicles or the formation of neutrophil extracellular traps (NETs) [3,4,5]. The fact that inflammatory and oxidative stress is implicated in the pathogenesis of cardiovascular diseases has triggered numerous investigations targeted to neutrophil-derived biomarkers, which may provide additional clinical benefit and allow early preventive treatment or intervention for cardiovascular patients [6].

In this review, we discuss the potential biomarker role of neutrophils and neutrophil-derived factors in abdominal aortic aneurysm (AAA) disease and elucidate relevant molecular mechanisms through which these cells are activated in inflammatory conditions. In addition, this review may be of interest to both basic scientists and clinicians to stimulate translational exchange and innovative clinical approaches.

## 2. Neutrophil Function in Cellular Defense

**Figure 1:**
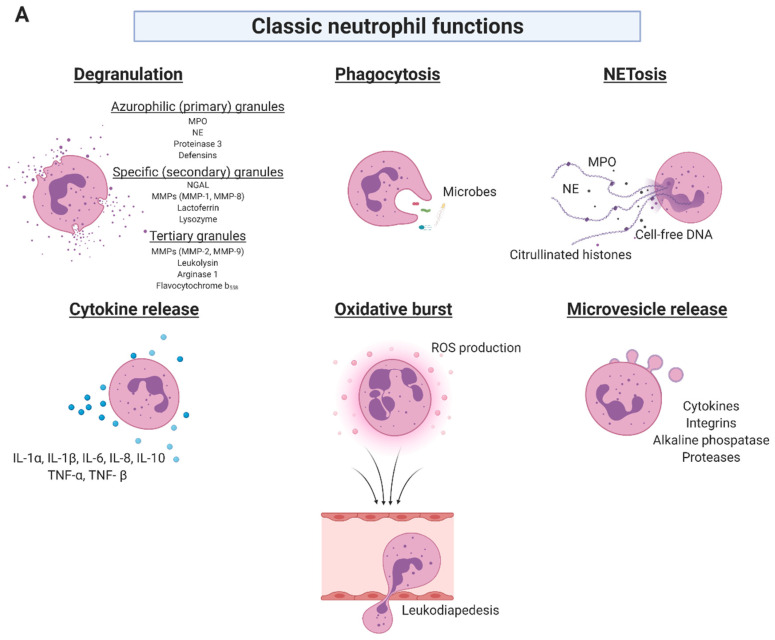

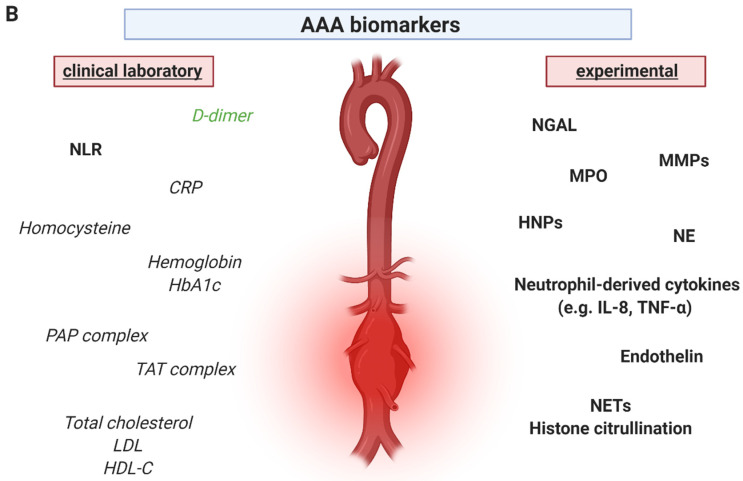
Overview of classical neutrophil functions as well as investigated AAA biomarkers. (**A**) Neutrophils are polymorphonuclear leukocytes that serve as the initial line of defense against invading pathogens and moreover play a key role in a variety of inflammatory conditions and cardiovascular diseases such as AAA. These granulocytes exert several classical functions, which can act as a double-edged sword of neutrophil antimicrobial function, but excessive activation can damage host tissue. Upon cell activation, several soluble neutrophil granule proteins are released. Phagocytosis and release of neutrophil extracellular traps are evolutionary conserved mechanisms of the unspecific immune defense, however, besides the desired antimicrobial functions, they may also contribute to the acute or chronic pathogenesis of vascular diseases. The production of cytokines by activated neutrophils is striking in its diversity. Neutrophils synthesize and release a variety of pro- and anti-inflammatory cytokines as well as growth factors. Additionally, neutrophils interact with surrounding immune cells and humoral factors leading to modification of cell recruitment and signal cascades via cytokine and microvesicle release. Triggered neutrophils activate their NADPH oxidase to generate substantial amounts of superoxide and other reactive oxygen species, which play a vital role in the progression of cardiovascular diseases. (**B**) In performing their functions, neutrophils provide a multitude of potential biomarkers, which can be used to improve the diagnosis and prognosis of AAA as well as postoperative outcome. To facilitate data presentation, this overview shows clinically available and experimental biomarkers, which have been investigated, but are currently not used in clinical practice. This selection presents neutrophil-derived biomarkers indicated in bold, clinical biomarkers in italics and specifically the most commonly applied clinical biomarker D-dimer in green. MPO: myeloperoxidase; NE: neutrophil elastase; NGAL: neutrophil gelatinase-associated lipocalin; MMP: matrix metalloproteinase; IL: interleukin; TNF-α: tumor necrosis factor-α; ROS: reactive oxygen species; AAA: abdominal aortic aneurysm; NLR: neutrophil-to-lymphocyte ratio; CRP: C-reactive protein; HbA1c: hemoglobin A1c; PAP: plasmin-alpha-2-antiplasmin; TAT: thrombin–antithrombin; LDL: low-density lipoprotein.; HDL: high-density lipoprotein; HNP: human neutrophil peptide; NET: neutrophil extracellular trap. Figure 1 was created by author J.K. with BioRender.com, last accessed on 8 August 2021.

Neutrophils represent 50–70% of circulating leukocytes in healthy adults and are typically the first effector cells at an inflammatory site, recruited to build the first line of immune defense to invading microbes [1,7]. In healthy adults, neutrophil production reaches up to 2 × 10^11^ cells per day controlled by granulocyte colony-stimulating factor and congenital neutropenia is associated with severe immunodeficiency in humans [8,9]. Neutrophils exert three primary effector functions: phagocytosis and generation of oxidative burst using ROS-dependent mechanisms and antimicrobial proteins such as lysozyme, lactoferrin, cathepsins and defensins which are intraphagosomally released (phagocytosis) or exocytosed into the extracellular space (degranulation). Furthermore, activated neutrophils can expel neutrophil extracellular traps (NETs)—net-like complexes composed of cell-free DNA, histones, and neutrophil granule proteins—in response to a variety of stimuli (Figure 1A) [2,3]. At the inflammatory site, neutrophils highly interact with surrounding immune cells and humoral factors, modifying cell recruitment and signal cascades [10].

Neutrophils produce various inflammatory as well as anti-inflammatory molecules, which are mainly released through their classified four different types of granules (Figure 1A) [7,11]. Primary, so-called azurophilic and peroxidase-positive granules, contain potent hydrolytic enzymes such as myeloperoxidase (MPO), neutrophil elastase (NE), proteinase 3, different types of defensins, cathepsin G, azurodicin, vitronectin and a mix of other bactericidal and endotoxin-neutralizing proteins [12,13,14,15,16,17,18]. While primary granule enzymes are principally responsible for killing, digesting and removing ingested microorganisms, secondary granules are known as specific, peroxidase-negative granules containing substances which may have regulatory functions outside the cell. These common effector molecules are lactoferrin, collagenases such as matrix metalloproteinase 1 (MMP-1) and MMP-8, lactoferrin, neutrophil gelatinase-associated lipocalin (NGAL), NADPH oxidase (NOX), M-ficolin, cysteine-rich secretory protein 3, cathelicidin LL-37 and lipocalin 2 [7,19,20]. Tertiary granules have overlapping contents with secondary granules, but predominately contain extracellular matrix (ECM) degrading proteins such as MMP-2 and MMP-9. In addition, these granules are filled with leukolysin, arginase 1 and flavocytochrome b_558_ [7,11,21,22]. Mature human neutrophils additionally contain easily mobilizable secretory vesicles that may be distinguished from azurophilic, specialized, and tertiary granules in terms of function. These secretory vesicles store preformed cytokines and deliver proteins to the cell surface that are required for cell adhesion, such as integrins, as well as alkaline phosphatase and proteases, to aid transmigration and immune protection [23,24,25]. Consequently, neutrophils and neutrophil-derived molecules represent a plethora of possible biomarkers upon neutrophil activation in cardiovascular diseases, which can be exploited to improve diagnosis, prognosis, as well as therapeutic options in the future.

## 3. Abdominal Aortic Aneurysms and Neutrophils

An abdominal aortic aneurysm is defined as an enlargement of the maximal aortic diameter to 30 mm or more [26]. Approximately 80% of AAA occur in the infrarenal abdominal aorta [27]. Men are more often affected by this disease than women, especially the elderly population [28]. An AAA is a multifactorial disease with environmental and genetic risk factors [29,30]. Apart from the non-modifiable risk factors gender, age and genetic predisposition, smoking is known as the most significant lifestyle factor. Other but less influential risk factors are hypertension, dyslipidemia, obesity, atherosclerotic occlusive disease, such as coronary artery disease, cerebrovascular disease or peripheral artery disease, and chronic obstructive pulmonary disease [31,32,33,34]. A rupture of the aortic vessel is an immediately life-threatening situation with a mortality of 85% to 90% outside a hospital [35]. The risk of rupture increases with AAA size and is about four times higher in women compared to men and doubles in smokers [36,37]. Hallmarks of AAA pathogenesis include: inflammation of the aortic wall leading to the infiltration of immune cells, including neutrophils, macrophages, B and T lymphocytes. This increases the activity of proteases and cytokines, which subsequently leads to weakening of the aortic wall, because extracellular matrix proteins, especially collagen and elastin, are destroyed. In addition, there is apoptosis of vascular smooth muscle cells. This weakening of the tunica media results in a vessel dilatation and forms the aneurysm [38,39]. A contributing issue in advanced AAA pathogenesis is the intraluminal thrombus (ILT), which occurs in about 75% of AAA patients and contains various proteases, in particular neutrophil-derived proteases [40,41,42]. Summarizing these pathological aspects, the AAA is nowadays mainly considered a chronic inflammatory disease with neutrophils recruited to the aneurysm site and into the ILT where they release detrimental constituents and substances, which contribute to the destruction of the aortic wall [43]. The AAA development and progression are characterized by an asymptomatic phenotype and varying growth rates associated with the risk of rupture, necessitating the need of reliable, widely available, and low-cost biomarkers [26]. Currently, there is no established medical treatment prior to AAA surgery and so far, no drug group or single drug has been sufficiently proven to be effective in reducing AAA growth [44]. According to the guidelines, the decision to perform surgery is generally based on three parameters: maximal AAA diameter greater than 50 mm in women and 55 mm in men; AAA expansion rate more than 10 mm per year; or symptomatic AAA [26,45]. When the indication for elective surgical intervention is given, there are currently two surgical treatment options available, open surgical repair (OSR) and endovascular aneurysm repair (EVAR) [46].

In clinical and laboratory studies, significant efforts were undertaken to identify circulating biomarkers for AAA and most of the examined potential biomarkers are involved in mechanisms of the immune system, hemostasis, ECM degradation or lipid metabolism (Figure 1B) [47,48]. Circulating molecules, such as D-dimer, fibrinogen, thrombin-antithrombin III-complex or C-reactive protein (CRP) have been reported to show higher levels in AAA patients [45,49,50,51]. As the most commonly applied clinical biomarker, D-dimer was also found to be positively correlated with AAA diameter, ILT size and AAA growth [52,53,54]. Other thrombosis-related biomarkers, such as plasmin-antiplasmin complex, homocysteine, and thrombin-antithrombin complex have also been linked to AAA expansion [55,56,57,58]. A meta-analysis reported on other dysregulated blood components such as MMP-9, interleukin (IL) 6, triglycerides, apolipoprotein A and low-density lipoprotein (LDL), but their clinical value remained unclear [59]. More recent studies and clinical trials are necessary to evaluate a potential biomarker value of these molecules. New insights suggest a more pronounced role of MMP-9 as a molecular marker for the evaluation of disease state and AAA rupture [60,61]. In the group of clinical laboratory biomarkers, the blood lipids, total cholesterol, LDL and high-density lipoprotein (HDL) showed associations with AAA pathogenesis and progression [62,63]. A study reported that body iron overload is not associated with AAA pathogenesis but demonstrated that the patient population had rather low preoperative and a postoperative increase of ferritin values [64]. This suggests that AAA pathogenesis is not directly related to iron metabolism, although iron deposits are described in AAA tissue and low levels of hemoglobin are associated with AAA [65]. An inverse relationship between the AAA expansion rate and HbA1c levels were discovered, suggesting that long-term high blood sugar may slow AAA progression in people with diabetes [66]. Of note, four retrospective analyses and one meta-analysis on metformin prescription to diabetic AAA patients revealed a significant reduction of AAA progression, rupture rate or incidence of repair when compared to AAA patients treated with other antidiabetic drugs. While the metformin effect ranged from 20% to 76% decrease in annual AAA growth, metformin consistently displayed a protective effect [67,68,69,70,71]. In addition, a preclinical experimental AAA model demonstrated the therapeutic efficacy of metformin to prevent aneurysm formation in normoglycemic mice with elastase induced AAA [67]. Currently, two European studies, the Swedish Metformin for Abdominal Aortic Aneurysm Growth Inhibition (MAAAGI) Trial (open-label randomized controlled trial) and the Vienna MetAAA Trial (a prospective, double-blind, randomized and placebo-controlled safety and efficacy study), which is initiated by our study team in Austria, test the hypothesis if metformin prescription could significantly limit AAA enlargement in nondiabetic patients [44,72,73].

Beside the above-mentioned clinical parameters, there is a multitude of experimental biomarkers described in the literature. Some of them are neutrophil-derived, that could be useful for the detection of AAA presence, prediction of AAA size, expansion rate, rupture risk, and postoperative outcome (Figure 1B). Neutrophils are highly implicated in the pathogenesis of AAA, driving the local inflammatory reaction including NET release as well as promoting ILT formation, mechanisms, which are harmful for the integrity of the aortic wall [74,75]. A study showed that after neutrophil depletion in mice, experimental AAA development is significantly inhibited with a decrease of both, tissue neutrophils and macrophages compared to the control group [76]. A Swedish case-control study reported on significantly higher blood counts of neutrophils, lymphocytes, monocytes, and basophils in AAA patients compared to healthy controls [77]. Neutrophils are recruited early, before monocytes, into the tissue then promoting monocyte extravasation by cytokine release, NET formation and granule protein secretion [78,79]. Interestingly, early reports stated that the amount of infiltrated inflammatory cells positively correlates with the size of the aneurysm and an increased expression and activity of MMP-2 and MMP-9 may contribute to rapid AAA growth and rupture of larger aneurysms [80]. The activity of NOX and superoxide production links oxidative stress to inflammation in AAA and is also correlated to aneurysm size [81]. This review was conducted to present the existing evidence of different neutrophil-derived factors that may be suitable to determine AAA presence, predict AAA size, expansion rate, rupture risk and surgical outcome. The neutrophil-derived factors assessed in the studies as well as their potential biomarker value are summarized in Table 1.

### 3.1. Neutrophil-to-Lymphocyte Ratio

The neutrophil-to-lymphocyte ratio (NLR) has been used as a subclinical inflammatory marker and has recently become evident as a useful predictor of malignancy, of prognosis in the coronavirus disease 2019 (COVID-19) as well as of cardiovascular risk and adverse outcomes [82,83,84,85]. A recent study assessed the role of NLR as a prognostic marker for AAA rupture. It was shown that the average NLR is significantly higher (9.3 vs. 3.39) in the patient group with ruptured AAA and that an NLR > 5 indicates a 5-fold increased risk of AAA rupture. In addition, AAA patients with NLR values > 5 also show significantly poorer outcomes in terms of 30-day mortality after OSR, regardless of ruptured or intact AAA [86]. Another study confirmed these results showing that an elevated NLR within 1 week after AAA surgery (OSR and EVAR) is strongly associated with postoperative complications and those patients may require closer follow-up [87]. Recently, it has additionally been reported that the preoperative NLR is a suitable long-term predictor for postoperative outcome after EVAR. In fact, AAA patients with elevated NLR before surgery show a significantly increased 5-year mortality as well as significantly higher 30-day, 1-year and 5-year reintervention rates after EVAR [88]. A meta-analysis of 4066 enrolled patients with aortic disease including AAA confirmed the positive association of an elevated NLR with AAA disease, higher aneurysm rupture risk, increased cardiovascular risk and mortality as well as higher postoperative reintervention rates. Indeed, it is not clear nor published in the current literature, if the NLR is able to predict AAA growth [89]. We compared the NLR of 39 AAA patients with 26 healthy controls and 39 patients with peripheral artery disease in an observational study (unpublished data). First, we found a significantly higher NLR in AAA patients (*p* = 0.026) and patients with peripheral artery disease (*p* = 0.019) compared to healthy controls. No significant difference was observed between the NLR of patients with AAA and patients with peripheral artery disease (*p* = 0.293). However, the NLR failed to predict aneurysm growth over the next 6 months regarding AAA and ILT (maximal diameter and volume) in a log-linear mixed model (unpublished data).

### 3.2. Neutrophil Gelatinase-Associated Lipocalin

As a neutrophil-derived protein, NGAL has recently been the subject of research in a variety of diseases and also seems to be a promising marker for the development and progression of AAA [90]. At the inflammatory site, neutrophils as major source of NGAL expression, promote the formation of NGAL/MMP-9 complexes and thus NGAL protects MMP-9 from proteolytic degradation and enhances its enzymatic activity. It was shown that NGAL/MMP-9 complexes were present in the ILT, the interface fluid and the aneurysm wall. The highest concentration of the NGAL/MMP-9 complexes was found in the luminal part of the thrombus (compared to abluminal and central ILT layers, aneurysm wall and interface fluid), if normalized to the tissue weight and total protein concentration. The general presence of NGAL/MMP-9 complexes in all ILT layers may lead to further ECM degradation of the adjacent vessel wall, promoting potential AAA growth [91,92]. Although the plasma levels of NGAL/MMP-9 complexes were found to be elevated in AAA patients, this biomarker did not correlate with the maximal aortic diameter or maximal ILT thickness [40,93]. According to another study, NGAL could be used as a surrogate marker for ILT biological activity and significantly reflects rather than predicts AAA growth [94]. Another aspect of how NGAL could be involved in aneurysm development is that it has been proven that c-Jun N-terminal-kinase is an important molecule in the pathophysiology of AAA, which upregulates both, NGAL and MMP-9 [95]. Of interest, it was previously described, that *Porphyromonas gingivalis* as most relevant pathogen in chronic periodontitis and associated periodontal bacteremia may contribute to AAA growth [96]. Since NGAL has bacteriostatic qualities, preventing bacteria from retrieving iron sources, it is possible that NGAL’s neutrophilic release in the iron rich ILT is a proteolytic response to accumulating bacteria in AAA tissue [97]. Regarding aortic rupture, an observational study revealed that blood concentrations of NGAL are significantly higher in patients with ruptured AAA compared to non-ruptured controls. In addition, comparing concentrations in human aneurysmatic wall tissue, there is a significantly higher NGAL expression in ruptured AAA compared to nondilated aortas, but no significant difference if compared to non-ruptured AAA [98]. A further study discovered serum and urine NGAL as renal predictors of acute kidney injury in AAA patients undergoing OSR [99].

Insights were also obtained with experimental AAA models, showing that either NGAL deficiency or anti-NGAL antibody blockade limits AAA expansion in mice. Analyses of deficient or treated mice also revealed significantly less neutrophil infiltration, diminished ECM degradation, decreased MMP activity and preservation of vascular smooth muscle cells [100]. Selective inhibition of c-Jun N-terminal-kinase targeting the upregulation of NGAL and degradation of the ECM lead not only to the prevention of AAA development, but also to regression of established AAA in two murine models [101].

Overall, NGAL has been demonstrated to be secreted by resident and circulating neutrophils in AAA patients, acting as a promising marker of AAA size and progression. Due to its role in AAA pathogenesis, where various substances target ECM integrity, NGAL appears to be a promising biomarker on which further investigation should be considered.

### 3.3. Neutrophil Elastase

Another neutrophil-derived protease that plays a crucial role in immune defense and ECM breakdown is neutrophil elastase. It is not only degrading elastin, but also collagen type III, IV and VI, fibronectin, laminin and distinct proteoglycans [102]. Neutrophil elastase further shifts the proteolytic balance in favor of ECM destruction by activating MMP-2, MMP-3, and MMP-9 and inactivating their inhibitor, tissue inhibitor of metalloproteinase-1 (TIMP-1) [103,104,105]. Other important NE effector functions are the activation of lymphocytes and platelets as well as the degradation of plasma proteins such as immunoglobulins, clotting factors or complement components. In addition, NE modifies lipoproteins, cleaves T-cell surface proteins and modulates the activity of cytokines such as IL-8, IL-1β and tumor necrosis factor-α (TNF-α) [102,106]. It is assumed that a local regulatory imbalance between NE and the endogenous protease inhibitor α1-antitrypsin is implicated in AAA pathogenesis. Despite growing knowledge and further research on the role of α1-antitrypsin deficiency in AAA, the currently available literature data is inconclusive [107].

It is commonly known that elastase release by neutrophils, either via degranulation or NETosis is implicated in the etiology of AAA [108]. Since NE increases inflammation and ECM breakdown in the aneurysmatic vessel wall, a direct link to aortic dilatation is evident. In the blood of AAA patients, NE was shown to be significantly higher compared to non-AAA controls. Furthermore, elastin-derived peptides induce NE release which was significantly higher in AAA patients compared to patients with aortic occlusive disease or healthy controls [108,109,110]. Interestingly, NE is highly associated with a negative impact on lung disorders such as emphysema [111]. Cigarette smoking, also well known as independent risk factor for AAA, augments the release of NE and its proteolytic activity [112,113]. A study compared the elastase activity between controls, AAA patients and patients with aortic occlusive disease according to their smoking behavior. The elastase activity level was greatest in the group of actively smoking AAA patients, followed by active smokers with aortic occlusive disease. Besides, there is a direct correlation between elastase activity and nicotine concentration [114]. Recently, studies showed that circulating NE-derived fibrin degradation products are elevated in patients with AAA and correlate with AAA and ILT volume as well as with the mechanical stress of the ILT [115,116]. The role of NE as a postoperative biomarker is controversial. A study analyzed blood samples of AAA patients undergoing OSR or EVAR for NE/α1-antitrypsin complexes and free elastase. Interestingly, after both surgical approaches, increased NE/α1-antitrypsin complexes were detected, whereas the free elastase levels decreased after OSR, but further increased after EVAR. Paradoxically, this may lead to a longer postoperative inflammatory response in patients undergoing less-invasive endovascular AAA surgery [117]. Another study revealed the opposite result, showing higher concentrations of NE/α1-antitrypsin complexes after OSR compared to EVAR, especially on the first day after surgery. Therefore, according to the results of this study, EVAR is linked to a postoperative reduction of the systemic inflammatory response. This indicates that NE may serve as neutrophil-derived biomarker for postoperative inflammatory response alterations [118].

Of note, in both angiotensin II and CaCl_2_-induced murine AAA models, NE-knockout mice had reduced aortic expansion or thoracic aortic dissection than wild-type littermates [119]. In line, another study using rats, feed with a novel, oral inhibitor of neutrophil elastase, showed significantly reduced AAA diameters, markers of neutrophil activation and elastase activity in the conditioned medium of AAA tissue [120].

### 3.4. Myeloperoxidase

Myeloperoxidase is a cationic heme-containing enzyme stored in primary azurophilic granules of neutrophils. When neutrophils are activated, MPO is released into the phagolysosomal environment as well as in the extracellular compartment [121]. At the infection site, controlled MPO release from neutrophils is critical for their role in host defense, but in contrast, uncontrolled degranulation exaggerates inflammation and can cause tissue damage. Myeloperoxidase-derived oxidants have been associated with serious chronic cardiovascular and liver diseases, diabetes mellitus and cancer. Thus, increased MPO activity is one of the most sensitive diagnostic tools for inflammatory and oxidative stress conditions [122].

Our recent study revealed a rather robust association of AAA disease and soluble MPO levels [54]. We showed that AAA patients almost have twice as high plasma MPO levels as compared to healthy controls. This was further confirmed in AAA wall tissue, verifying indeed the AAA site as source of MPO, with more than eleven times higher MPO concentrations than in healthy aortas. Moreover, the plasma and tissue-released MPO levels significantly correlated with the maximal AAA diameter [54]. Another study discovered that higher baseline MPO concentrations are significantly associated with faster AAA progression, independent of the aortic baseline diameter. With regard to AAA growth, a prognostic value of MPO with 80% sensitivity and 59% specificity was yielded [123]. We further developed a combined score of the clinical biomarker D-dimer and the experimental marker MPO and found it to be beneficial in AAA diagnosis and prognosis [54]. The two parameters combined in a diagnostic score reached a sensitivity of 73% and specificity of 80%. In addition, regarding the prognostic marker value, the combined score outperformed D-dimer alone by identifying patients with rapid AAA growth (≥2 mm over the next 6 months) with 72% sensitivity and 67% specificity. Overall, D-dimer and MPO seem to be two sensitive biomarkers of AAA that reflect separate pathomechanistic aspects of AAA disease. While D-dimer is considered a robust clinical marker for AAA, albeit non-specific, it is mostly related to ILT presence and size. Myeloperoxidase may allow detection and monitoring of AAA irrespective of thrombus presence, as it is reflecting the central inflammatory character of AAA pathogenesis [54]. However, currently it is not clear whether MPO especially contributes to aneurysm rupture in the abdominal aorta. Comparably, a study on cerebral aneurysms suggests that histological MPO presence positively correlates with 5-year aneurysm rupture risk [124]. However, there are significant differences in etiology and pathomechanism between cerebral and aortic aneurysms. Further studies on AAA rupture and perioperative risk are needed to clarify whether MPO can potentially be used as a biomarker of AAA instability.

### 3.5. Neutrophil Extracellular Traps

Another feature of neutrophils are NETs, which are released structures composed of chromatin DNA, citrullinated histones and granular proteins such as NE, MPO, cathepsin G and gelatinase [2,125]. These NETs are an evolutionary conserved innate immune response to pathogen infection, but they also seem to play a key role in the etiology of cardiovascular diseases such as atherosclerosis, AAA, and thrombosis, as well as in autoimmune, metabolic, malignant, and infectious diseases [3,126,127]. It was revealed that NETs are formed at early stages (2 to 3 days after aneurysm induction) of experimental AAA in mice [126,128]. In line, applying DNase I as anti-NET therapy suppresses aneurysm formation in experimental AAA mouse models [128,129]. While DNase I targets the DNA that serves as the backbone of NETs, chloro-amidines were tried successfully as an alternative method to block histone citrullination in a mouse model, leading to NET reduction and subsequent considerably decreased aneurysm formation [126]. In this review, expelled components of these NETs, such as MPO or NE are already identified and discussed as potential biomarkers in the pathophysiology of AAA. Besides, various NET constituents are currently under research to function as novel AAA biomarker.

Citrullinated histones and cell-free DNA were found to be elevated in the plasma and tissue of AAA patients [96,126,128]. Our recently reported study focused on NET-specific histone citrullination and biomarker analysis of citrullinated histone H3 (citH3) in AAA disease [130]. Plasma levels of citH3 were found to be considerably higher in AAA patients compared to healthy controls and remained significant for AAA presence in multivariable analysis. Regarding the prognostic marker value, with 77% sensitivity and 64% specificity, baseline citH3 levels exceeding 194 ng/mL indicated fast aneurysm expansion (≥2 mm increase over 6 months in computed tomography angiography determined AAA diameter). Furthermore, normalization of plasma citH3 levels after surgical AAA repair (in both, OSR or EVAR) was observed. Of note, further expansion of an established experimental AAA was prevented in mice treated with a NET inhibitor targeting histone citrullination. Therefore, it seems that citH3 represents a promising AAA biomarker and histone citrullination a potential therapeutic target to control AAA disease progression [130]. We and others found the highest concentration of NET components in the adventitia, but also the ILT showed depositions of citrullinated histones [126,128,130].

Thus, neutrophil extracellular traps are clearly associated with AAA pathogenesis and risk. Further studies are necessary to determine their biomarker specificity since NETs are also found in the context of other diseases.

### 3.6. Neutrophil-Associated Cytokines and Chemoattractants

Neutrophil activation and vascular inflammation lead to a distinct pro-inflammatory and chemotactic cytokine microenvironment of the AAA wall. A study discovered 15 significant differences in the cytokine expression profile comparing human AAA tissue and non-aneurysmal controls. Several pro-inflammatory cytokines such as IL-1α, IL-1β, IL-6, IL-8, TNF-α, TNF-β, and oncostatin M as well as the anti-inflammatory cytokine IL-10 were upregulated in AAA tissue compared to non-aneurysmal controls [131]. Early study reports already showed significantly elevated plasma concentrations of IL-1, IL-2, IL-6, IL-8 and TNF-α in AAA patients compared to healthy individuals. Additionally, higher levels of TNF-α were shown in asymptomatic AAA patients compared to patients with either symptomatic AAA or aneurysm rupture [132,133,134,135]. We recently showed that IL-1β, which was reported to trigger NET formation, was found to be significantly increased in tissue-conditioned medium of AAA patients, but not in the tissue-conditioned medium of ILT compared to aortas from transplant donors [130]. Neutrophil chemotaxis to the inflammatory AAA site is highly regulated by potent chemoattractants such as IL-8 [48]. A study reported that IL-8-positive staining is associated with neutrophil presence at the luminal pole of the ILT. Moreover, the ILT displays a negative gradient from the luminal to the abluminal layer, with the luminal layer releasing considerably more IL-8. The aneurysmal media and adventitia also release IL-8, however, released IL-8 concentrations by ILT were found to be fourfold higher when compared to the aortic wall [40]. A study of more than 350 enrolled AAA patients assigned participants to groups depending on the AAA diameter to small (<45 mm), medium (45–55 mm) and large (>55 mm). It was not only reported that plasma IL-6 concentrations of AAA patients are significantly increased compared to healthy controls, but also that plasma IL-6 levels increase with AAA size [136]. Another study presented a mathematical model based on the serum concentration of IL-6 that predicts AAA growth, but the clinical relevance remains to be elucidated [137]. It was demonstrated that plasma IL-10 remains significantly positively correlated with the annual AAA expansion rate in a multivariate analysis of 386 AAA patients after correcting for putative aneurysm confounding factors and baseline AAA diameters [138].

Particularly in patients with ruptured AAA, studies suggest an increase of pro-inflammatory cytokines such as IL-6, IL-8, and TNF-α in either plasma or aortic tissue extracts [139,140,141]. In contrast, AAA patient plasma or explanted AAA lesion culture frequently shows reduced levels of anti-inflammatory cytokines such as IL-10, also especially in patients with ruptured AAA [140,142,143]. This significantly altered cytokine profile in AAA supports the detrimental disease state and associated rupture risk. Nevertheless, there are conflicting results, which report increased IL-10 plasma levels in ruptured AAA patients compared to non-ruptured AAA patients [141]. It is unclear if these contradictory findings resulted from the small size of the study population or from differences between patients or study designs. However, neutrophils are significant producers of IL-10. It is proposed that rupture of an AAA activates the inflammatory system with a compensatory anti-inflammatory response. Anti-inflammatory IL-10 is positively correlated with pro-inflammatory cytokines such as IL-6, IFN-γ, and IL-17A, and also CRP has been shown to support the proposed compensatory anti-inflammatory response of IL-10 in AAA patients [138,141,144].

Recently, a systematic review compared variations of circulating cytokine levels and differences of post-interventional inflammatory responses after OSR and EVAR [145]. Seventeen studies identified a significantly greater systemic inflammatory response after OSR with elevated cytokine levels, especially of IL-6 and IL-8. On the other hand, some studies yielded contradictory results or showed no differences between the two surgical approaches regarding levels of IL-1β, IL-10, and TNF-α [145]. Another study demonstrated a post-interventional normalization of the inflammatory state. Reasonably high preoperative serum levels (50–100 pg/mL) of IL-1α and IL-8 significantly dropped six months post-EVAR [146]. Of note, there is a significant positive correlation of preoperative IL-1α serum concentrations and preoperative AAA size. In addition, the decrease in serum IL-1α level post-EVAR and the recruitment of inflammatory neutrophils also showed a significant positive correlation. The observed significant reduction of IL-1α and concomitant decrease in neutrophil recruitment after AAA surgery implies that this cytokine might be a marker of successful surgical outcome [146].

Animal studies have also highlighted the relevance of various cytokines in AAA pathogenesis. Infiltrating neutrophils are a major source of IL-1β, which induces NET formation and promotes AAA [126]. A study revealed significantly elevated IL-1β mRNA and protein levels in abdominal aortas after experimental AAA induction in mice. Furthermore, IL-1β neutralization, either through genetic deletion or pharmacologic receptor antagonism, attenuates experimental AAA initiation and formation, which was shown in two different AAA mouse models [147,148]. The inhibition of TNF-α via intravenous administration of tumor necrosis factor binding protein protected against AAA formation in an elastase-induced rat model [149]. Likewise, double knockout mice with cluster of differentiation 4 and interferon-γ deficiency did not develop aneurysms in the CaCl_2_-induced murine AAA model. Conversely, after intraperitoneal IFN-γ administration, aneurysm development was partially reconstituted [150].

The chronic inflammatory nature of the aortic aneurysmal vessel wall and pathogenic implications of cytokines are central to AAA disease. Obtaining a complete picture of the cytokine expression in AAA is difficult. Many studies focus solely on a narrow repertoire of molecules, typically due to technical constraints. This may miss unique and unexpected features in the cytokine profile of AAA disease [131].

### 3.7. Matrix Remodeling Proteases and Their Inhibitors

The most characteristic histopathological factor of AAA is extracellular matrix degradation and remodeling, especially of collagen and elastin, which subsequently leads to weakening of the aortic wall [38,39]. Human AAA data show that proteases, mainly MMPs, cysteine proteases and serine proteases, derived from neutrophils, macrophages or smooth muscle cells play key roles in ECM degradation and AAA formation [151]. Although there is also a physiological fluctuation of MMP activity in healthy aortic tissue, in the aneurysmal aortic wall, MMPs primarily degrade elastin and collagen. The most common metalloproteinases in human AAA tissue are MMP-1, MMP-2, MMP-3, MMP-9, MMP-12, MMP-13 with different substrate preferences. For the regulation of ECM composition and as an inhibitory role against most of the known MMPs, TIMP-1 and TIMP-2 are also expressed [2,151,152,153]. Gelatinase B, another name for MMP-9, seems to have a crucial role, because it was shown, that targeted gene disruption of MMP-9 in mice suppresses the development of experimental AAA [152,154]. Furthermore, it was revealed that both MMP-2 and MMP-9 are necessary to induce experimental AAA formation in mice [155]. At the inflammatory site, MMP-9 also promotes revascularization by activating vascular endothelial growth factor. Cysteine proteases, such as cathepsin S, cathepsin K, cathepsin D and cathepsin L have an impact on ECM degradation as well as serine proteases such as plasmin, tissue plasminogen activator or urokinase plasminogen activator have the ability to activate MMPs [2,151,152,153].

Matrix metalloproteinase 9 seems to be the most abundant metalloproteinase expressed in AAA, as its mRNA levels are more than 20 times and 2 times higher than transcripts of MMP-1 and MMP-2, respectively [156]. Based on a comprehensive meta-analysis, it was discovered that circulating MMP-9 concentrations are higher in AAA patients than in controls without AAA. Therefore, it was suggested that higher circulating MMP-9 concentrations are linked to the presence of AAA [157]. In line, another study reported that higher concentrations of MMP-9 and IL-6 were associated with future risk of clinically diagnosed AAA, independent of the established AAA risk factors [158]. Of note, MMP-9 expression was shown to be correlated with AAA diameter (more than 50 mm), indicating a role for this MMP in later stages of AAA disease [159]. There is further evidence, that besides large ILT size and high aortic wall stress, elevated plasma concentrations of the circulating biomarker MMP-9, are associated with increased AAA growth rates [160]. Additionally, the MMP-9 concentration in the adjacent aneurysmal aortic wall correlates with the ILT thickness [161]. At the aneurysmal rupture site, it was demonstrated that levels of MMP-8 and MMP-9 were significantly increased compared to concentrations of anterior wall biopsies of the same AAA patients [162]. Furthermore, a study showed an association between preoperative elevation of plasma MMP-9 concentrations and non-survival at 30 days from rupture surgery and therefore it was suggested as survival indicator [163].

In addition to MMP-9, various other neutrophil-derived MMPs are elevated and support the pathogenic process in aneurysm development [159]. It was reported that MMP-1 is significantly increased in AAA tissue compared to aortic tissue from organ transplant donors and elevated plasma MMP-1 levels are associated with increased rates of AAA rupture and reduced survival [163,164]. In the context of a genetic predisposition to an elevated MMP-2 expression, it has been speculated that MMP-2 is central in AAA development and may be regarded as a candidate gene for AAA formation [165]. Despite the fact that elevated levels of MMP-2 have been discovered in AAA tissue, systemic MMP-2 levels could not be applied to predict the expansion of small AAA, possibly due to limited levels reaching circulation [166]. Mice deficient in MMP-3 present with reduced ECM damage and lower susceptibility to AAA development. Conversely, MMP-3 overexpression and activity aggravate experimental AAA [167]. While MMP-7 shows increased expression in AAA and weakens the aortic wall via the promotion of smooth muscle cell apoptosis, the MMP-13 concentration in human aortic tissue was likewise found to be 1.8-fold higher in AAA compared to atherosclerotic aortas and was not expressed in aortic tissue of organ transplant donors [168,169]. In both, developing and ruptured AAA tissue, the evaluation of protease activity revealed a threefold increase of MMP-8, a fivefold increase in the cysteine proteases K and L, and a 30-fold increase in cathepsin S activation compared to aortic tissue of organ transplant donors. Importantly, the abundance of MMP-8 was confirmed in growing and ruptured AAA by immunohistochemistry; MMP-8 was found stored in the secondary granules of neutrophils, i.e., mostly expressed by infiltrating neutrophils [170]. After experimental induction of AAA in mice, a mean increase of 63% in aortic diameter was reported for wildtype mice, while MMP-12 deficient mice only increased at a mean of 26% [171]. An additional study involving another murine AAA model showed that MMP-12 was required for AAA progression and rupture [172].

Endogenous TIMPs inhibit the excessive proteolytic ECM destruction of MMPs. The balance of MMPs and TIMPs is critical in vascular remodeling and angiogenesis. An imbalance in the MMP and TIMP activity ratio is likely involved in the etiology of vascular disorders such as AAA [173]. In an elastase-induced murine AAA model, TIMP-1 knockout mice develop bigger aneurysms than corresponding control mice, suggesting TIMP-1 to have a protective effect on AAA formation and growth [174]. Interestingly, AAA expansion and rupture are prevented if MMP activity is inhibited by local overexpression of TIMP-1 in a rat xenograft model [175]. The expression of TIMP-1 in human AAA tissue has been demonstrated to correlate with both, MMP-2 and MMP-9 [176]. Moreover, the MMP/TIMP ratio has been shown to be altered in AAA patients compared to controls [163]. Although the plasma TIMP-1 concentrations of AAA patients were significantly higher than that in healthy controls, it was found that the TIMP-1 levels in AAA wall tissue were considerably lower than that in healthy aortic tissue [177,178]. Two studies with 18 and 163 AAA patients, respectively, investigated TIMP-1 as yearly AAA growth marker and did not yield significant outcomes [179,180]. A study analyzing fatal AAA rupture demonstrated that patients with AAA rupture have higher plasma levels of TIMP-1. Logistic regression analysis revealed that higher TIMP-1 levels were an independent predictor of fatal AAA rupture [179]. In contrast, another study suggests (without providing statistical significance) that patients with AAA rupture, especially if fatal, may have lower preoperative TIMP-1 plasma concentrations compared to non-ruptured AAA patients or survivors of AAA rupture [163]. A study using TIMP-2 deficient mice showed significantly reduced aneurysm development after experimental AAA induction. Since this is a rather surprising and counterintuitive result, it possibly can be explained by the role of TIMP-2 as a cofactor in MMP-2 activation. Thus, determining the net effect of TIMP-2 on matrix degradation and AAA formation is difficult [181]. In addition, it is known that also TIMP-3 has an important regulatory function in AAA pathogenesis, especially in preventing adverse vascular remodeling. A deficiency of TIMP-3 deteriorates AAA pathology [182]. Unfortunately, due to a paucity of clinical evidence in the existing literature on these pathophysiologically important TIMPs, most of these proteins are not known for diagnosing asymptomatic AAA or predicting AAA growth.

### 3.8. Human Neutrophil Peptides

Neutrophils produce a large amount of human neutrophil peptides (HNPs), which are important constituents of the innate immune system and released after neutrophil activation, most abundantly HNP1, HNP2, and HNP3 (also known as α-defensins) [183,184]. While HNPs are scarcely detectable in circulation of healthy humans, these peptides are increased in the plasma of AAA patients and detectable within the ILT, with a positive gradient from the abluminal to the luminal pole. Interestingly, a study showed significant positive correlations between α-defensins and NGAL/MMP-9 complexes, neutrophil elastase and MPO, but only α-defensins significantly correlated with maximal ILT thickness [40,185].

### 3.9. Endothelin

Endothelin is a potent vasoconstrictor derived from the endothelium that modulates the vascular tone of human arteries and veins. A range of variables, including angiotensin II, vasopressin, thrombin, hypoxia, shear stress and vascular damage, induce its release and several of these processes are involved in the pathophysiology of AAA [186]. Endothelin enhances the adhesion of neutrophils to endothelial cells and mediates neutrophil transmigration [187]. An experimental mouse study demonstrated that endothelin-1 overexpression exacerbates atherosclerosis and induces aortic aneurysms via lowering high-density lipoprotein and increasing inflammatory cell infiltration, oxidative stress, and MMP-2 levels in the adjacent perivascular fat, the vascular wall and in atherosclerotic lesions [188].

Human plasma endothelin levels were found to be highly and significantly elevated in AAA patients versus controls. Patients with large aneurysms (≥50 mm) displayed higher concentrations than patients with small AAA (<50 mm). However, the levels of endothelin-1 and 2 did not statistically differ between symptomatic and asymptomatic AAA patients [189]. A study with 178 AAA patients and a total number of 491 follow-up years reported that endothelin-1 and baseline AAA diameter persisted as predictors of AAA growth above median (2.5 mm per year) in logistic regression analysis [190]. Surprisingly, it was revealed that patients undergoing OSR of ruptured infrarenal AAA with fatal postoperative organ failure have significantly lower levels of endothelin-1 compared to successfully operated and surviving AAA patients. This suggests that elevated circulating endothelin concentrations may also be an acute reaction and safeguarding response to hemorrhagic AAA rupture, with ensuing systemic ischemia and reperfusion. Thus, low endothelin levels in this group of individuals may be an early symptom of severe and irreversible whole-body hypoperfusion [191].

**Table 1:** Summary of discussed study characteristics on potential neutrophil-derived biomarkers in AAA.

Neutrophil-Derived Factor	Diagnosis	Prognosis	Surgical Outcome	Rupture
**Neutrophil-to-lymphocyte ratio**	↑ NLR associated with AAA disease, increased rupture risk, elevated cardiovascular risk, and mortality as well as higher postoperative reintervention rates [89]	NLR failed to predict AAA and ILT growth over the next 6 months (unpublished data, n.s.)	NLR > 5 showed higher 30-day mortality after OSR [86] ↑ NLR within 1 week after OSR or EVAR associated with postoperative complications [87] preoperative ↑ NLR increased 5-year mortality and 30-day, 1-year, 5-year reintervention rates after EVAR [88]	↑ NLR (9.3 vs. 3.39) in patients with ruptured compared to intact AAA [86] NLR > 5 indicated a 5-fold increased risk of AAA rupture [86]
**Neutrophil gelatinase-associated lipocalin**	highest concentrations of NGAL/MMP-9 complexes were found in the luminal part of the ILT (compared to abluminal and central ILT layers, aneurysm wall and interface fluid) [91,92] surrogate marker for ILT biological activity [94]	reflects rather than predicts AAA growth [94]	serum and urine NGAL as renal predictors of acute kidney injury in AAA patients undergoing OSR [99]	↑ NGAL blood concentrations in ruptured AAA patients (compared to non-ruptured controls) [98] ↑ NGAL expression in tissue of ruptured AAA compared to nondilated aortas [98]
**Neutrophil elastase**	↑ NE blood levels in AAA patients compared to non-AAA controls [108,110] elastin-derived peptides induced NE release in AAA patients > aortic occlusive disease > healthy controls [108,109,110] ↑ circulating NE-derived fibrin degradation products in AAA patients correlated with AAA and ILT volume and ILT mechanical stress [115,116]		↑ NE/α1-antitrypsin complexes after OSR and EVAR; ↓ free elastase levels after OSR, but ↑ after EVAR [117] ↑ NE/α1-antitrypsin complexes only after OSR compared to EVAR, especially on the first day after surgery; EVAR was linked to a reduced postoperative systemic inflammatory response [118]	
**Myeloperoxidase**	AAA patients: two times higher plasma MPO levels compared to healthy controls and more than eleven times higher MPO concentrations in aortic tissue [54] plasma and tissue-released MPO levels correlated with the maximal AAA diameter [54] MPO combined with D-dimer reached in a diagnostic score a sensitivity of 73% and specificity of 80% [54]	↑ baseline MPO concentration was significantly associated with faster AAA progression, independent of aortic baseline diameter [123] MPO combined with D-dimer in a prognostic score outperformed D-dimer alone by identifying patients with rapid AAA growth (≥2 mm over the next 6 months) with 72% sensitivity and 67% specificity [54]		
**Neutrophil extracellular traps**	↑ citrullinated histones and cell-free DNA in the plasma and tissue of AAA patients [96,126,128] ↑ citH3 plasma levels in AAA patients compared to healthy controls [130] highest concentration of NET components in the adventitia, depositions of citrullinated histones in the ILT [126,128,130]	baseline citH3 levels exceeding 194 ng/mL indicated fast aneurysm expansion (≥2 mm diameter increase over 6 months in CTA) as prognostic marker value, with 77% sensitivity and 64% specificity [130]	normalization of plasma citH3 levels after OSR and EVAR [130]	
**Neutrophils and associated cytokines**	↑ plasma concentrations of IL-1, IL-2, IL-6, IL-8 and TNF-α in AAA patients compared to healthy individuals [132,133,134,135] pro-inflammatory (IL-1α, IL-1β, IL-6, IL-8, TNF-α, TNF-β, oncostatin M) and anti-inflammatory (IL-10) cytokines were upregulated in AAA tissue compared to non-aneurysmal controls [131] ↑ IL-1β levels in tissue-conditioned medium of AAA patients (but not of ILT) compared to aortas from transplant donors [130] ILT displayed a negative IL-8 gradient from the luminal to the abluminal layer, IL-8 was associated with neutrophil presence at the luminal pole of the ILT, released IL-8 concentrations by ILT were fourfold higher compared to the aortic wall (media and adventitia) [40]	plasma IL-6 levels increased with AAA size [136] serum concentration of IL-6 predicts AAA growth in a mathematical model, but clinical relevance remains to be elucidated [137] plasma IL-10 positively correlated with the annual AAA expansion rate [138]	seventeen studies identified a significantly ↑ systemic inflammatory response after OSR with ↑ cytokine levels, especially of IL-6 and IL-8; some studies yielded contradictory results or showed no differences between OSR and EVA regarding levels of IL-1β, IL-10, and TNF-α [145] post-interventional normalization of the inflammatory state, preoperative serum levels (50–100 pg/mL) of IL-1α and IL-8 significantly dropped six months post-EVAR [146] preoperative IL-1α serum concentrations correlated with AAA size, serum IL-1α levels and neutrophil recruitment decrease post-EVAR [146]	↑ TNF-α levels in asymptomatic AAA patients compared to patients with either symptomatic AAA or aneurysm rupture [132,133,135] ↑ pro-inflammatory IL-6, IL-8, and TNF-α levels in plasma and aortic tissue extracts of ruptured AAA patients [139,140,141] ↓ anti-inflammatory IL-10 levels in AAA patient plasma or explanted AAA lesion culture [140,142,143] Conflicting results report ↑ IL-10 plasma levels in ruptured compared to non-ruptured AAA patients, may be a compensatory anti-inflammatory response [141]
**Matrix metallo-proteinases and their inhibitors**	MMP-1, MMP-2, MMP-3, MMP-9, MMP-12, MMP-13 are most common in AAA tissue [2,151,152,153] MMP-2 as a candidate gene for AAA formation [165] ↑ MMP-2 levels in AAA tissue, but systemic MMP-2 levels cannot predict the expansion of small AAA [166] ↑ aortic tissue MMP-7 expression and associated smooth muscle cell apoptosis [168] most abundant MMP-9 mRNA levels 20 and 2 times higher expressed than MMP-1 and MMP-2 transcripts, respectively [156] circulating MMP-9 levels were linked to AAA presence and ↑ MMP-9 concentrations in AAA patients compared to controls [157] ↑ MMP-9 and IL-6 levels were associated with future risk of developing AAA [158] MMP-9 expression correlated with AAA diameter (>50 mm diameter) and ILT thickness [159,161] ↑ MMP-1 levels in AAA tissue compared to organ transplant donors [164] 1.8-fold ↑ aortic tissue MMP-13 concentration in AAA compared to atherosclerotic aortas, but no expression in tissue of organ transplant donors [169] ↑ plasma TIMP-1 concentrations in AAA patients compared to healthy controls, but ↓ TIMP-1 levels in AAA wall compared to healthy aortic tissue [177,178]	plasma MMP-9 concentrations were associated with increased AAA growth rates, larger ILT and high aortic wall stress [160]	↑ preoperative plasma MMP-9 levels were associated with non-survival at 30 days from rupture surgery, MMP-9 as survival indicator [163]	↑ plasma MMP-1 levels were associated with increased rates of AAA rupture and reduced survival [163] ↑ MMP-8 and MMP-9 levels at the aneurysmal rupture site compared to anterior wall biopsies of the same AAA patients [162] A threefold ↑ of MMP-8 activity, a fivefold ↑ in the cysteine proteases K and L and a 30-fold ↑ in cathepsin S activation in developing and ruptured AAA compared to organ transplant donors [170] immunohistochemical MMP-8 abundance in growing and ruptured AAA [170] ↓ preoperative TIMP-1 plasma concentrations in (fatal) ruptured AAA patients compared to survivors or non-ruptured AAA patients (n.s.) [163] ↑ plasma TIMP-1 levels in AAA patients served as predictor of fatal AAA rupture [179]
**Human neutrophil peptides**	↑ plasma levels in AAA patients detectable within the ILT: luminal > abluminal, correlated with maximal ILT thickness [40,185]			
**Endothelin**	↑ levels identified large (≥50 mm) vs. small (<50 mm) AAA [189]	↑ levels predict AAA growth above median (2.5 mm per year) [190]	↓ levels in ruptured, but successfully operated (OSR) and surviving AAA patients compared to patients with fatal postoperative organ failure [191]	

Abbreviations: NLR, neutrophil-to-lymphocyte ratio; AAA, abdominal aortic aneurysm; ILT, intraluminal thrombus; n.s., not significant; OSR, open surgical repair; EVAR, endovascular aneurysm repair; NGAL, neutrophil gelatinase-associated lipocalin; MMP, matrix metalloproteinase; NE, neutrophil elastase; MPO, myeloperoxidase; citH3, citrullinated histone H3; CTA, computed tomography angiography; IL, interleukin; TNF, tumor necrosis factor; TIMP, tissue inhibitor of metalloproteinase; ↑, increased; ↓, decreased.

## 4. Current Perspective and Future Prospects

Neutrophils and their derived molecules, as components of the innate immune system, play a vital role in the prevention and treatment of infections. Furthermore, they may act as central pathogenic trigger in cardiovascular diseases with an inflammatory component, such as AAA. Neutrophil-derived biomarkers may therefore offer a reasonable approach for the detection of AAA and prediction of AAA expansion in the future. The existing literature is abundant, sometimes with contradictory results, making it difficult to draw definite conclusions for clinical application. Numerous circulating substances in the peripheral blood may be suitable to determine AAA presence, predict AAA size, expansion rate, rupture risk, and postoperative outcome. Only a few of them have the potential to be clinically relevant and to be used in clinical trials in the future. There are significant limitations due to the fact that many investigated biomarkers linked to AAA prognosis are not disease specific, considering their well-established association with underlying atherosclerosis, hemostatic processes (ILT occurrence), inflammation and NET formation. Beside the plethora of investigated biomarkers, it is challenging to compare heterogeneous study designs, small and diverse study populations, and methodological experimental setups as well as varying AAA imaging techniques. Currently, D-dimer is the most commonly applied clinical biomarker for AAA, but clinicians are still confronted with a major absence of powerful and accurate tools to ensure the best monitoring and outcome of AAA patients. It seems that a combination of clinical and experimental biomarkers, such as D-dimer and MPO reflects distinct components of AAA pathomechanism and this may yield an improved score and validity for AAA diagnosis and prognosis. Considering the particular need for prognostic biomarkers (since currently no established medical treatment exists prior to AAA surgery), confounder and validation studies should be pursued based on encouraging explorative data for neutrophil-derived biomarkers.

## Data Availability

Not applicable.

## Abbreviations

AAA	Abdominal aortic aneurysm
ADAMTS	A disintegrin and metalloproteinase with thrombospondin motifs
citH3	Citrullinated histone H3
CRP	C-reactive protein
ECM	Extracellular matrix
EVAR	Endovascular aneurysm repair
HDL	High-density lipoprotein
HNP	Human neutrophil peptide
IL	Interleukin
ILT	Intraluminal thrombus
LDL	Low-density lipoprotein
MMP	Matrix metalloproteinase
MPO	Myeloperoxidase
NE	Neutrophil elastase
NET	Neutrophil extracellular trap
NGAL	Neutrophil gelatinase-associated lipocalin
NLR	Neutrophil-to-lymphocyte ratio
NOX	NADPH oxidase
OSR	Open surgical repair
ROS	Reactive oxygen species
TIMP	Tissue inhibitor of metalloproteinase
TNF-α	Tumor necrosis factor-α
